# Financial Reasons for Working beyond the Statutory Retirement Age: Risk Factors and Associations with Health in Late Life

**DOI:** 10.3390/ijerph191710505

**Published:** 2022-08-23

**Authors:** Denise Burkhalter, Aylin Wagner, Sonja Feer, Frank Wieber, Andreas Ihle, Isabel Baumann

**Affiliations:** 1 Institute of Public Health, ZHAW Zurich University of Applied Sciences, 8400 Winterthur, Switzerland; 2 Swiss Paraplegic Research, 6207 Nottwil, Switzerland; 3 Chair of Social Psychology and Motivation, University of Konstanz, 78464 Konstanz, Germany; 4 Center for the Interdisciplinary Study of Gerontology and Vulnerability, University of Geneva, 1205 Geneva, Switzerland; 5 Department of Psychology, University of Geneva, 1205 Geneva, Switzerland; 6 Swiss National Centre of Competence in Research LIVES—Overcoming Vulnerability: Life Course Perspectives, 1015 Lausanne, Switzerland

**Keywords:** older workers, working life prolongation, reasons for retirement, socioeconomic inequality, public health, public policy reform

## Abstract

Despite an increasing trend of working life prolongation, little is known about the risk factors for financial reasons for working beyond the statutory retirement age (SRA), and how these reasons relate to health. The present study examined (1) the determinants of working beyond the SRA, (2) the workers’ self-reported reasons for working beyond the SRA, and (3) the association between these reasons and health in late life. Cross-sectional data of 1241 individuals from the Swiss survey “Vivre/Leben/Vivere” were analyzed. The results showed that people with a low level of education and with a low income have an 80% higher risk of working beyond the SRA for financial reasons than for other reasons (*p* < 0.001). Moreover, self-rated health was not significantly associated with working beyond the SRA for financial reasons but was significantly associated with education and income (*p* < 0.01). In conclusion, while previous studies have already identified financial difficulties as one important reason for working beyond the SRA, the present study indicated the socioeconomic factors that are crucial for increasing the risk for working beyond the SRA. Thus, our results help to guide the adaptation of social policies for better maintaining and promoting the health of particularly vulnerable older workers.

## 1. Introduction

While early retirement was promoted in many OECD countries until the 1990s, these policies were reversed thereafter due to financial deficits in old-age pension systems [1]. Currently, most countries incite a prolongation of working life, for instance, by raising the statutory retirement age (SRA) [2,3]. The trend of working life prolongation is, however, not only observed in countries and periods where the SRA has been increased, but as shown by the case of Switzerland, also in contexts without such increases. Although SRA has not been raised since 2005, the labor force participation rate of workers above the age of 65 in Switzerland has steadily increased since then (except during the COVID-19 pandemic, when labor force participation among older workers stagnated) [4,5]. In other words, older workers prolong their working lives, although they are not constrained to do so by raises in the SRA, as in other countries. The case of Switzerland therefore provides an interesting laboratory to study the additional drivers of working life prolongation besides raises in SRA.

Switzerland has a labor force participation rate of older workers that is high in international comparison. This phenomenon has been attributed to the social security system and to the situation in the labor market that is characterized by generally low levels of unemployment and a shortage of skilled labor [6]. Characteristics of the social security system that contribute to the high labor force participation rate are the contribution rates to occupational pension plans that increase with age. As employers pay large shares of these contributions, older employees are incited to remain in the labor force. The Swiss pension system consists of three pillars [6]: (1) public old-age and survivors’ insurance (AHV/AVS), (2) occupational pension plans, and (3) private pension provision. The first pillar is compulsory, has a strongly redistributive character (i.e., redistributes pension benefits from high-income to low-income workers), and aims at covering the basic living costs of retirees. The second pillar insures employees with an income above a certain threshold and aims at assuring the accustomed living standard. The third pillar consists of voluntary, tax-deducible, private savings. In addition, supplemental benefits may be claimed if the income from the other pillar(s) is not sufficient to cover the basic living costs [7].

Retirement is not mandatory at the SRA, but the SRA is a requirement for full pension benefit eligibility (in case of retirement before the SRA, there is an actuarial adjustment, i.e., a reduction in pension benefits) [7]. The SRA is 65 for men. The SRA for women was 62 until 2001. In 2001, the retirement age for women of birth cohorts 1939–1941 was raised to 63. In 2005, the retirement age for women of the birth cohorts younger than 1941 was raised to 64. Research on this pension reform showed that women adjusted to these policy changes by working for longer. The effect was stronger for the younger cohorts and for less affluent women [8]. Accordingly, women in our sample who belong to these younger cohorts may have been incited to prolong their working life. Retiring after the SRA thus implies that these younger cohorts of women worked until a later age than the older cohorts of women in our sample.

Furthermore, some of the cohorts included in our study (1931–1946) were affected by the financial crisis of 2008/2009 while still in employment. The crisis may have hampered their labor force participation; for instance, through layoffs and generally adverse labor market conditions for older workers that were observed during and after the financial crisis [9,10]. At the same time, potential layoff during the crisis and subsequent gaps in the old-age pension savings may have forced some of the workers in our sample to continue working beyond the SRA.

On an aggregated level, three key drivers have contributed to the international trend of prolonging working lives: first, increases in life expectancy, second, structural changes of the economy and society—such as an increasingly female and highly educated workforce—and third, changes in labor market and old-age pension policies [3]. Changes in the labor market and old-age pension policies consisted of a scaling back of measures that pushed or pulled workers into early retirement schemes [2]. Furthermore, they consisted of measures to actively incite working life prolongation. Such measures were categorized in the academic literature as MAINTAIN and NEED factors. MAINTAIN factors consist of incentives for firms to maintain older workers (e.g., wage subsidies for older workers or age-friendly workplace measures), whereas NEED factors consist of incentives for workers to remain in the workforce (e.g., raises in retirement age or cuts in the level of public pensions) [2,11]

However, individual retirement intentions are the result of complex processes that may not align with the intentions of public policies. On an individual level, the academic literature thus distinguishes between STAY and STUCK factors to designate older workers’ motives to work beyond the SRA [12]. STAY factors consist of job-related aspects that contribute to voluntarily prolongations of working life (e.g., an interesting job or social contacts at the workplace). STUCK factors consist of job-related aspects that contribute to involuntarily prolongations of working life (e.g., financial reasons or lack of a successor) [12,13,14]. Such STUCK factors have been found to be more prevalent in countries with low levels of public pensions [15,16], and among workers with no or low levels of occupational and private pension savings, low levels of education and low levels of income among those employed in manual jobs and those who experienced career interruptions—typically women [17,18,19,20]. One of these studies used the same dataset as in the present study, but distinguished between voluntary and involuntary retirement, whereas the present study uses a more fine-grained categorization of the reasons for working beyond the SRA [19].

The distinction between voluntary and involuntary retirement, or between more fine-grained reasons for working beyond the SRA, highlights that retirement timing is influenced by multiple factors at the micro-, meso- and macro-level. In addition to workers’ own retirement intentions, the rules and expectations of employers and country-level policies influence the timing [15,21]. Accordingly, individuals who retire early tend to be fundamentally different from those who retire late [22]. This selection bias results in the healthy worker effect, where healthier workers tend to prolong their working life, while less healthy workers tend to retire early [23,24].

While the drivers of STUCK factors, and among them, the financial reasons for working beyond the SRA, have been studied in the past, little is known about how such financial reasons affect people’s health. To the best of our knowledge, the only study on this topic is a Dutch study that examined the relationship between bridge jobs and older people’s life satisfaction. The authors compared people who took up bridge jobs with those who did not, and compared people who indicated different reasons for taking up a bridge job [25]. The study found that taking up a bridge job for financial reasons—which can be categorized as STUCK factor—led to a decrease in life satisfaction as compared to motives that can be categorized as STAY factors [25]. In contrast, a study examining the impact of policy-based increases in the SRA—which can be categorized as NEED factors—on older people’s job satisfaction, does not find significant effects [26]. Nevertheless, such policy changes seem to be negatively related to mental health; in particular, if increases in SRA are experienced a few years before retirement [27,28,29]. Furthermore, a recent quasi-experimental study from Spain has shown that a policy change to remove an early retirement scheme had a detrimental effect on workers’ mortality between the ages 60 and 69, particularly affecting low-skilled workers in physically and psychosocially demanding jobs [30].

Given that our knowledge for the reasons working beyond the SRA—especially for STUCK factors—and its relationship to health is incomplete, this study aimed at investigating, first, the determinants of working beyond the SRA, second, the workers’ self-reported reasons for working beyond the SRA and its determinants, and third, the association between reasons for working beyond the SRA and self-reported health. This approach allowed us to better understand which socioeconomic groups are particularly prone to remain in the labor force—be it for STAY or STUCK reasons—and which socioeconomic groups are particularly vulnerable to working beyond the SRA for STUCK reasons.

Based on the literature, the following hypotheses were tested: H1: Financial reasons for working beyond the SRA (relative to other reasons) are more common among women than men (H1a), more common among those with lower levels of education level (H1b), more common among those in manual occupations than those in non-manual occupations (H1c), and more common among those with lower levels of income (H1d). H2: Health status is worse among persons who have reported financial reasons for working beyond the SRA than among persons who reported other reasons.

To test these hypotheses, we relied on a unique survey, a representative study of people above age 65 in Switzerland (n = 1241) that assessed the reasons for working beyond the SRA based on an open-ended question. This survey is one of the few studies that assessed the reasons for working beyond the SRA. Unlike other surveys that used a closed-ended question in the questionnaire to assess the reasons [31,32], the survey we used included an open-ended question. To the best of our knowledge, our study is the first to provide an analysis of a detailed assessment of the reasons for working beyond the SRA.

This article is structured as follows: After presenting the materials and methods, our analysis proceeds in three steps: First, we examine the participants’ characteristics and the determinants of retirement timing. Second, we analyze the reasons for retiring after the SRA and its determinants. Third, we scrutinize the relationship between the reasons for retiring after the SRA and health. Finally, we discuss our results and sum up with a conclusion.

## 2. Materials and Methods

### 2.1. Study Design and Data

In this study, cross-sectional data from the Swiss survey “Vivre/Leben/Vivere” (VLV) from 2011 were used [33]. VLV aims to survey the health and living conditions of the Swiss population aged 65 and over, and it comprises a sample of 3080 persons. The sample is representative of the Swiss population aged 65 years and older and was conducted in three languages (German, French, and Italian) in five Swiss cantons. In the present study, we included persons aged 65 to 80 years to ensure that among those who worked beyond the SRA, no more than 15 years had passed between the time of retirement and the interview (reducing the sample to 1833 individuals, or 59.5% of the original sample). Regression analyses were based on a complete case analysis (i.e., only persons with no missing data in the variables used in the regression analyses were included) (reduction in the overall sample to 1241 individuals, or 40.3% of the original sample, and to 82 cases for workers who worked beyond the SRA).

### 2.2. Measures

#### 2.2.1. Outcomes

Work beyond the SRA was assessed by means of a self-reported measure on retirement timing, distinguishing between before, at, and after the SRA. The SRA generally refers to 64 for women and 65 for men. Among women, the birth cohorts before 1939 had an SRA of 62, and the birth cohorts for 1939–1941 had an SRA of 63. For certain occupational groups (e.g., pilots), companies, or economic sectors, the statutory retirement age is lower than the regular retirement age [6]. Among respondents with missing information at the self-reported measure on retirement timing, those who reported to be in employment at the moment of the survey and those who reported not to be in retirement were coded as retiring after the SRA.

Participants were asked in an open-ended question about the reasons for working beyond the SRA. We coded the open-ended answers into four categories of working beyond the SRA: 1 = financial reasons, 2 = offer/desire of previous employer, 3 = self-employment/new job, 4 = personal/social reasons. The variable, “reason for working beyond the SRA” was also used as an independent variable in this study. In case participants named more than one reason, only the first mentioned reason (i.e., the main reason) was coded and assigned to the classification.

Self-rated health at the time of the survey was measured on a 5-point Likert scale (1 = poor, 2 = rather poor, 3 = satisfactory, 4 = good, 5 = very good). Robustness analyses were performed with other health status variables (e.g., depression, nervousness/stress, or various chronic diseases such as diabetes), but the results did not differ substantially from results with the variable “self-rated health”.

#### 2.2.2. Covariates

Socioeconomic and sociodemographic variables included gender (1 = female, 2 = male), education level (1 = less than secondary education, 2 = secondary education: apprenticeship and high school, 3 = tertiary education: technical college and university), gross monthly household income in Swiss francs (CHF) (1 = < 6000, 2 = 6000–10,000, 3 = > CHF 10,000), and occupational group. The thresholds of the gross monthly household were based on information on average pensions and they income considered the fact that many people live with a partner. More precisely, the average gross monthly wage in 2011 was CHF 7100 [5]. The monthly gross pension for a male with an average pre-retirement earning was CHF 5304 in 2013 (own calculation based on the net replacement rate of average pre-retirement earnings of 74.7% provided by OECD Statistics [5]). Occupation was coded according to the 2008 International Standard Classification of Occupations (ISCO-08) and then divided into three groups: 1 = higher non-manual occupations (ISCO-08 groups 1–3), 2 = lower non-manual occupations (ISCO-08 groups 4–6), 3 = manual occupations (ISCO-08 groups 7–9). In the regression analyses, we controlled for age and age squared.

### 2.3. Statistical Analyses

In a first step, the data were analyzed descriptively to present the descriptive statistics of the overall analytic sample and of the specific group of workers retiring after the SRA. In a second step, the determinants of working beyond SRA were examined using a multinomial regression analysis. In a third step, we univariately examined the reasons for retiring after the SRA, indicating the share of workers in each category. In a fourth step, we conducted multinomial regression analyses using “financial reasons” as the reference category to assess the association between the reasons for working beyond the SRA and sex, occupation, education, and income, adjusted for age and age squared. Subsequently, the predicted probabilities were calculated, indicating the probability in percentage points and the 95% confidence intervals. As a robustness test, a logistic regression with a binary dependent variable (“financial reasons” vs. “all other reasons”) was calculated. The results were not substantially different from the multinomial logistic regression. In a fifth step, an ordinary least square (OLS) regression was calculated to assess the association between self-rated health and the reasons for retirement after the SRA, adjusted for sex, occupation, education, income, age and age squared. The analyses were conducted using the STATA software (version 17). For the multinomial logistic regression analysis, we used the command “mlogit”, for the OLS regression analysis the command “reg”.

## 3. Results

### 3.1. Participants’ Characteristics

Table 1 presents the descriptive statistics for the total analytic sample which consisted of 1241 participants. Furthermore, Table 1 presents the descriptive statistics of the specific group of workers retiring after the SRA (6.6% of the total sample; 49.4% retired at the SRA, 44% retired before the SRA). In the total sample, respondents were 72.5 years old at the time of the survey (*SD* = 4.4). There was a total of 42.8% women and 57.2% men. A total of 14.1% of the respondents had less than secondary education, 58.3% had a secondary education, and 27.6% had a tertiary education. A total of 32.8% of respondents were in a higher non-manual occupation, 48.2% were in a lower non-manual occupation, and 19% were in a manual occupation. A total of 43.2% of respondents had a monthly gross household income of less than CHF 6000, with 47.6% between CHF 6000 and 10,000, and 9.2% with more than CHF 10,000.

Table 2 presents the results of the determinants of retiring after the SRA as compared to retiring at or before the SRA. Men were significantly more likely to retire before the SRA, as compared to at the SRA for women (*p* < 0.001). Workers with an income of >CHF 6000 were significantly more likely to retire before the SRA as compared to at the SRA, than workers with an income <CHF 6000 (*p* = 0.001; *p* < 0.001). Workers in lower non-manual and manual occupations were significantly less likely to retire after the SRA, as compared to at the SRA, than higher non-manual workers.

### 3.2. Reasons for Working beyond the SRA 

Table 3 shows the identified reasons for working beyond the SRA. With a total of 30%, personal and social reasons were reported most frequently. A total of 28% of participants stated that they had received an offer or had worked beyond retirement age at the request of their previous employer, and 23% stated that they had worked longer for financial reasons. Self-employment or starting a new job was mentioned least frequently (17%).

### 3.3. Factors Associated with Working beyond the SRA 

**Figure 1:**
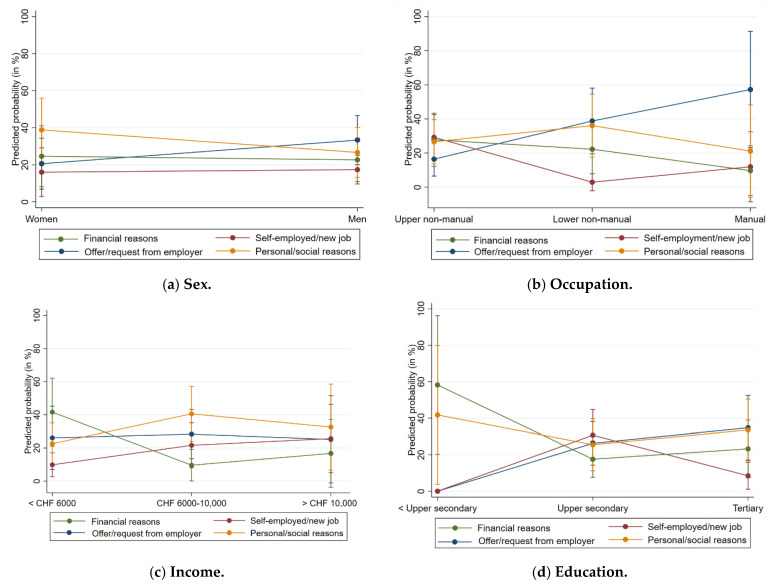
(**a**–**d**) Predicted probabilities and 95% confidence intervals, based on a multinomial logistic regression on reason for working beyond the SRA.

The multinomial logistic regression analyses on the reasons for working beyond the SRA (for full results, see (Table S1)) showed no significant differences between genders (Figure 1a), occupational groups (Figure 1b), and income levels (Figure 1c). Only education levels differed, such that persons who had less than secondary education (Figure 1d) reported “financial reasons” as often as “personal/social reasons”, but both of these reasons were mentioned more frequently than “employer offer/desire” and “self-employed/new job” reasons. Thus, individuals with a low education were more likely to retire later, due to financial or personal/social reasons, than due to an offer or request from the employer, self-employment, or a new job.

**Figure 2:**
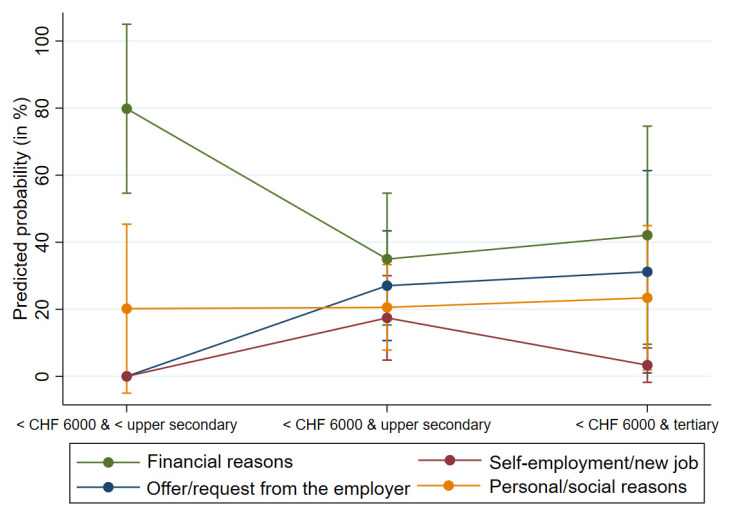
Predicted probabilities and 95% confidence intervals for the lowest level of income and for all levels of education, based on a multinomial logistic regression on the reason for working beyond the SRA.

When the predicted probabilities for several independent variables were calculated together, and specific values were again distinguished (e.g., a low, medium, or high level of education), financial reasons for working beyond the SRA were significantly more likely than other reasons—with a predicted probability of around 80%—for persons with a gross monthly income of less than CHF 6000 and a low level of education (less than secondary education), with zero to 20% (Figure 2). The results of the multinomial regression analysis can be found in Table S1.

### 3.4. Factors Associated with Self-Rated Health

An OLS analysis for self-rated health showed that there was no significant relationship between the reason for working beyond the SRA and self-rated health (see Table 4). There was a significant association between self-rated health, education level, and income class.

## 4. Discussion

Based on the Swiss VLV data, the present study examined the determinants of working beyond the SRA, self-reported reasons for working beyond the SRA and its determinants, and the association with self-rated health. A total of 6.6% of the respondents worked beyond the SRA. Workers in higher non-manual occupations were more likely to work beyond the SRA than other occupational groups. Based on a first hypothesis (H1), we investigated whether women, persons with low levels of education, in manual occupations, and with low levels of income are more likely to work beyond the SRA for financial reasons. The analysis showed that of these factors, when considered as a net effect, only the level of education level made a significant difference. Individuals with a low level of education were more likely to work beyond the SRA, due to financial or personal/social reasons, rather than due to an offer or request from an employer, self-employment, or a new job. However, when different factors were considered together, it could be shown that individuals who *simultaneously* have a low level of education *and* a low level of income are significantly more likely to work beyond the SRA for financial reasons. Thus, H1 was partially confirmed.

The present study thus adds to the literature on financial reasons for working beyond the SRA, by highlighting the risk factors for working beyond the SRA for STUCK reasons. While previous studies identified financial difficulties as an important reason for working beyond the SRA [12,20,34,35,36], this study was able to show which risk factors favor work beyond the SRA due to financial difficulties. Accordingly, we provide evidence for the risk factors for STUCK reasons. Gender was not a predictor. Earlier studies had shown that working beyond the SRA is particularly common among individuals without occupational pension benefits, which, in Switzerland, affects women significantly more often than men [14,37]. A possible reason for this difference could be that in the present study, the regression analysis for household income included the income of the partner, whereas in the other studies, the individual financial situation (especially retirement savings and financially difficult periods in the life course) was considered.

While our study, as well as previous studies, finds that lower educated workers are more prone to prolonging their working life due to STUCK reasons, this fate does not seem to be easily anticipated. A study examining the adjustment of the expected retirement age after an increase in the SRA in Germany in 2007 showed that although younger workers overall expected to work longer than in previous generations, those with lower levels of education adjusted their expectations to a lesser extent [38]. The authors maintain that the slower updating of retirement expectations among this group of workers causes concern regarding their income security after retirement.

A second hypothesis (H2) asked whether the health status of people who reported financial reasons for working beyond the SRA is poorer than that of people who reported other reasons. The analysis showed that there was no significant relationship between the reason for working beyond the SRA and self-rated health. Thus, H2 was rejected. A possible explanation of this result is the reduction of a possible cognitive dissonance, i.e., a reduction of a contradiction between one’s own actions and value orientations, as individuals are motivated to perceive one’s actions in line with one’s values [39]. In order to reduce such a cognitive dissonance—that one works although one’s health status is poor—one’s own health status could be assessed as being better than it actually is. Nevertheless, it is noteworthy that education and income were significantly associated with both the reasons for working beyond SRA and health status. This suggests that a low level of education and a low level of income seem to constitute a common risk factor [12].

An alternative explanation for the missing association between the reason for working beyond the SRA and self-rated health may be that the result was influenced by the reforms of the old-age pension system in 2001 and 2005. These reforms led to an increase in women’s SRA from 62 to 63 for birth cohorts 1939–1941, and to 64 for the birth cohorts younger than 1941. For the older cohorts of women, working beyond the SRA thus meant that they were still relatively young in the first year of working beyond the SRA (i.e., 63). Working beyond the SRA may have been less detrimental to their health as compared to those women who were 64 or 65 when working beyond the SRA, and as compared to men who were 66 when working beyond the SRA. Our findings of the association between health and low levels of education and income may be explained by the social gradient in working life prolongation among the cohorts of women affected by the policy change. As previous research has shown, not all women adjusted to the raise in SRA in the same way: less affluent women were more likely to retire at the new SRA of 63 (birth cohorts 1939–1941) and 64 (birth cohorts younger than 1941), while more affluent women tended to continue retiring at the former SRA of 62 [8].

The limitations of the study are, first, the small sample size of late retirees. The results of the regression analyses must therefore be considered to be exploratory. The share of people working beyond the SRA assessed in our study was lower than in other datasets such as OECD Statistics, which indicated for Switzerland for the year 2011 that approximately 20% of workers aged 65–69 and approximately 10% of workers aged 70–74 were in paid employment [5]. The potential reasons for these differences were that our sample encompassed people older than age 74, an age group among which labor force participation was lower. Furthermore, our analysis included only individuals that reported the reasons for working beyond the SRA, which was not the case with all survey participants. Nevertheless, despite the small sample size, our study substantially contributes to the understanding of older people’s reasons for working beyond the SRA. In contrast to reasons for early retirement, which are assessed in largely exploited panel studies such as the Survey of Health, Ageing and Retirement in Europe [16,36,40], only few surveys assess the information on the reasons to working beyond the SRA [31,32]. A second limitation of our study is that health status was not measured immediately after retirement, but at the time of the survey, a time when respondents had already been retired for up to 15 years, as it has been performed in a previous study using the same dataset [19]. Thus, the short-term effects of working beyond the SRA for financial reasons on health status may have already faded. Our results thus represented mid- to long-term effects. A third limitation is that the reasons for early retirement are self-coded based on open-ended answers. This poses a certain risk for inaccuracy, partly because multiple reasons were given in some cases. Fourth and finally, given that selection bias probably affects our results, the study does not allow for causal conclusions.

## 5. Conclusions

This study showed that the phenomenon of working life prolongation was observed also in contexts without raises in SRA. While many workers continued to work for STAY reasons, some also continued for STUCK reasons. The latter seemed particularly frequent among workers with a low level of education and income. Since older workers with low levels of education and low levels of income were also at a higher risk of health problems [41], special attention should be addressed to these vulnerable groups of older workers.

One way to address vulnerable groups of workers may be to improve the working conditions for those who work beyond the SRA. This option consists, for instance, of providing workers who continue working beyond the SRA with additional time to recover, for instance, a right to a continuously increasing number of vacations by age, through a statutory right to work part-time, or through flexible retirement schemes, as known by other countries [21,42,43]. In a recent study, the latter has shown to have positive effects on the health of older people prolonging their working lives [30]. These measures may benefit not only the most vulnerable groups of workers, but may additionally provide an incentive for older workers in general to remain in the labor force beyond the SRA. Another approach for addressing the most vulnerable workers may be to guarantee that public old-age pensions cover a minimal living standard for everyone and that access to supplemental benefits for those in need is facilitated.

Directions for future research are first to examine the influence policy changes on working life prolongation, for instance, to examine how health among women of different birth cohorts is affected by raises in SRA. A second direction for research may be to examine the development of financial reasons for retirement after the SRA longitudinally over the past two decades in Switzerland.

## Figures and Tables

**Table 1:** Participant characteristics.

	Total (100%, *N* = 1241)		Retirement after the SRA (6.6%, *n* = 82)	
Variables	%	N	%	N
Sex				
Women	42.8	531	37.8	31
Men	57.2	710	62.2	51
Education level				
<Secondary education	14.1	175	7.3	6
Secondary education	58.3	723	53.7	44
Tertiary education	27.6	343	39.0	32
Occupational groups				
Higher non-manual	32.8	407	50.0	41
Lower non-manual	48.2	598	36.6	30
Manual	19.0	236	13.4	11
Household income				
<6000 CHF	43.2	536	42.7	35
6000–10,000 CHF	47.6	591	43.9	36
>10,000 CHF	9.2	114	13.4	11
Self-rated health				
Poor	1.0	12	1.2	1
Rather poor	5.6	69	6.1	5
Satisfactory	31.5	389	23.2	19
Good	48.5	598	56.1	46
Very good	13.5	166	13.8	11
Age in years, mean (*SD*)	72.5 (4.4)		73.3 (3.8)	

CHF, Swiss francs, SD, standard deviation.

**Table 2:** Determinants of retiring after the statutory retirement age (SRA).

Ref = Retirement at the SRA	Retirement before the SRA		Retirement after the SRA	
Variables	Coeff.	SE ^a^	Coeff.	SE ^a^
Gender (ref = women)				
Men	0.496 ***	(0.140)	0.189	(0.259)
Education level (ref = < secondary education)				
Secondary education	0.028	(0.191)	0.580	(0.455)
Tertiary education	−0.325	(0.231)	0.700	(0.503)
Occupational groups (ref = higher non-manual)				
Lower non-manual	0.115	(0.159)	−0.578 **	(0.280)
Manual	−0.271	(0.199)	−0.765 *	(0.396)
Income (ref = < 6000 CHF)				
6000–10,000 CHF	0.822 ***	(0.138)	0.061	(0.272)
>10,000 CHF	0.805 ***	(0.240)	0.303	(0.416)
Age	−0.138	(0.208)	−0.036	(0.364)
Age^2^	−0.0004	(0.0003)	0.0001	(0.0005)
Constant	1.572	(1.179)	−2.795	(2.072)
N	1241			
Pseudo R-square	0.057			

Ref, reference category, CHF, Swiss francs, SE, standard errors. ^a^ Robust SE were used, *** *p* < 0.001, ** *p* < 0.01, * *p* < 0.05.

**Table 3:** Reasons for working beyond the SRA.

Reasons	%	N
*Financial reasons (total)*	*23.0*	*19*
Financially necessary	10.0	8
Supplement pension	13.0	11
*Offer/request from employer (total)*	*28.0*	*23*
Being needed in the workplace	26.0	21
Skills are valued in the workplace	2.0	2
*Self-employed/new job (total)*	*17.0*	*14*
Self-employment	13.0	11
Started new position	4.0	3
*Personal/social reasons (total)*	*30.0*	*26*
Good health	2.0	2
Partner still working	1.0	1
Importance of social contacts	1.0	1
Likes the work	24.0	20
Employment is good for you	1.0	1
Has found a new challenge	1.0	1

**Table 4:** OLS regression for self-rated health.

	Self-Rated Health	
	Coef.	SE ^a^
Reason for working beyond the SRA (ref = financial)		
Offer/request from employer	−0.099	(0.283)
Self-employment/new job	0.118	(0.297)
Personal/social	0.110	(0.236)
Gender (ref = women)		
Men	0.055	(0.189)
Education level (ref = < secondary education)		
Secondary education	0.919 **	(0.330)
Tertiary education	1.295 **	(0.385)
Occupation groups (ref = higher non-manual)		
Lower non-manual	0.222	(0.216)
Manual	−0.110	(0.334)
Income (ref = < 6000 CHF)		
6000–10,000 CHF	−0.817 **	(0.239)
>10,000 CHF	−0.237	(0.221)
Age	−0.443	(0.311)
Age^2^	0.000	(0.000)
Constant	3.209 *	(1.567)
*N*	82	
R^2^	0.333	

Ref, reference category, CHF, Swiss francs, *SE*, standard errors. ^a^ Robust SE were used, ** *p* < 0.01, * *p* < 0.05.

## Data Availability

The data presented in this study are publicly available at the SwissUbase repository of the Swiss Centre of Expertise in the Social Sciences (FORS), https://www.swissubase.ch/de/, project reference number 10685, accessed on 29 June 2022.

## Supplementary Materials

The following supporting information can be downloaded at: www.mdpi.com/article/10.3390/ijerph191710505/s1, Table S1: Multinomial logistic regression on reason for working beyond the SRA.
