# Supplementary material for: Financial Reasons for Working beyond the Statutory Retirement Age: Risk Factors and Associations with Health in Late Life
Source: Int J Environ Res Public Health. 2022 Aug 23;19(17):10505. doi: 10.3390/ijerph191710505 (PMC9518211; doi:10.3390/ijerph191710505)
Supplement: Supplementary file 1 [file ijerph-19-10505-s001.zip › ijerph-1819944-supplementary.pdf]

Supplementary Materials

Table S1. Multinomial logistic regression on reason for working beyond the SRA.

	Reason for working beyond the SRA (ref = financial)					
	Offer/request from employer		Self-employment/new job		Personal/social	
	Coef.	*SE*[a]	Coef.	*SE*[a]	Coef.	*SE*[a]
Gender (ref = women)						
Men	0.784	(0.960)	0.311	(1.008)	−0.290	(0.857)
Education level (ref = secondary education)						
No or compulsory education	−16.17***	(1.298)	−16.29***	(1.612)	−0.987	(1.077)
Tertiary education	−0.058	(0.901)	−2.017*	(0.935)	−0.054	(0.859)
Occupational groups (ref = lower non-manual)						
Higher non-manual	−1.156	(1.003)	2.307	(1.244)	−0.559	(0.897)
Manual	1.755	(1.536)	2.812	(1.815)	0.455	(1.278)
Income (ref = 6,000-10,000 CHF)						
< 6,000 CHF	−1.784	(1.180)	−2.781*	(1.110)	−2.254*	(0.949)
> 10,000 CHF	−0.711	(1.305)	−0.327	(1.335)	−0.841	(1.130)
Age	−3.208*	(1.537)	−2.509	(1.595)	−0.415	(1.294)
Age^2	0.004*	(0.002)	0.004*	(0.002)	0.002	(0.002)
Constant	−13.16	(7.185)	−16.71*	(7.753)	−6.884	(6.142)
N			82			
Pseudo R-square			0.232			

Ref = Reference category, CHF = Swiss francs, SE = Standard errors

[a] Robust standard errors were used, *** $p < 0.001$, * $p < 0.05$
